# Predictive and Prognostic Significance of mRNA Expression and DNA Copies Aberrations of *ERCC1*, *RRM1*, *TOP1*, *TOP2A*, *TUBB3*, *TYMS*, and *GSTP1* Genes in Patients with Breast Cancer

**DOI:** 10.3390/diagnostics12020405

**Published:** 2022-02-04

**Authors:** Matvey M. Tsyganov, Marina K. Ibragimova, Evgeniy Yu. Garbukov, Irina A. Tsydenova, Kseniya A. Gaptulbarova, Daria S. Dolgasheva, Ekaterina A. Zdereva, Anastasia A. Frolova, Elena M. Slonimskaya, Nikolai V. Litviakov

**Affiliations:** 1 Cancer Research Institute, Tomsk National Research Medical Center of the Russian Academy of Sciences, 634050 Tomsk, Russia; tsyganovmm@yandex.ru (M.M.T.); jrmaximum@rambler.ru (E.Y.G.); tsydenova422@gmail.com (I.A.T.); xenia.gaptulbarova@yandex.ru (K.A.G.); normikus.18.97@gmail.com (D.S.D.); zdereva.e@gmail.com (E.A.Z.); anastasiya10152@gmail.com (A.A.F.); slonimskaya@rambler.ru (E.M.S.); nvlitv72@yandex.ru (N.V.L.); 2 National Research Tomsk State University, 634050 Tomsk, Russia

**Keywords:** chemotherapy’s gene expression, copy number aberrations, neoadjuvant chemotherapy, efficiency, prognosis

## Abstract

Increasingly, many researchers are focusing on the sensitivity in breast tumors (BC) to certain chemotherapy drugs and have personalized their research based on the assessment of this sensitivity. One such personalized approach is to assess the chemotherapy’s gene expression, as well as aberrations in the number of DNA copies—deletions and amplifications with the ability to have a significant effect on the gene’s activity. Thus, the aim of this work was to study the predictive and prognostic significance of the expression and chromosomal aberrations of eight chemosensitivity genes in breast cancer patients. Material and methods. The study involved 97 patients with luminal B breast cancer IIB–IIIB stages. DNA and RNA were isolated from samples of tumor tissue before and after treatment. Microarray analysis was performed for all samples on high-density microarrays (DNA chips) of Affymetrix (USA) CytoScanTM HD Array and Clariom™ S Assay, human. Detection of expression level of seven chemosensitivity genes—*RRM1*, *ERCC1*, *TOP1*, *TOP2a*, *TUBB3*, *TYMS*, and *GSTP1*—was performed using PCR real-time (RT-qPCR). Results. The expression of the *RRM1* (AC scheme), *TOP2α*, *TYMS*, and *TUBB3* genes in patients with an objective response to treatment (complete and partial regression) is higher than in patients with stabilization and progression (*p <* 0.05). According to our results, the presence of a high level of *GSTP1* in a tumor biopsy is associated with the low efficiency of the NAC CP scheme (*p =* 0.05). The presence of *RRM1* deletion is associated with complete and partial regression, as for the *TOP1* and *TUBB3* genes (*p <* 0.05). Higher rates of metastatic survival are associated with a high level of expression and amplification of the *GSTP1* gene (log-rank test *p =* 0.02 and *p =* 0.05). Conclusion. Thus, a complex assessment of the chemotherapy’s gene expression is important not only for understanding the heterogeneity and molecular biology of breast cancer but also to obtain a more accurate disease prognosis.

## 1. Introduction

The most important aspect of personalized treatment of cancer patients is the resistance and sensitivity to specific chemotherapeutic drugs [1]. For this purpose, it is possible to determine markers of chemosensitivity in tumor tissue. Thus, many studies have shown that the expression and/or co-expression of several genes, such as *ERCC1*, *RRM1*, *TOP1*, *TOP2α*, *TUBB3*, *TYMS*, and *GSTP1*, in tumor tissues is closely related to chemoresistance and prognosis in breast cancer patients (BC) [2]. It was found that the *ERCC1* gene (excisional repair gene) is a structure-specific endonuclease involved in DNA repair. Clinical studies have shown that high *ERCC1* expression is associated with resistance to platinum-based chemotherapy [3], as well as overexpression of glutathione-S-transferase P1 (*GSTP1*), which belongs to the family of metabolic enzymes, which is involved in the detoxification of some anticancer drugs by conjugating with glutathione [4], which is also associated with low efficacy of chemotherapy based on anthracyclines and taxanes, as well as low rates of disease-free and overall survival [4,5].

Thymidylate synthase (*TYMS*) and ribonucleotide reductase (*RRM1*) are involved in the de novo formation of thymidylate and dNTP from ribonucleotides, respectively. The high expression of *TYMS* and low *RRM1* significantly correlate with sensitivity to gemcitabine [6]. *TUBB3* is a marker for docetaxel and paclitaxel resistance. The high expression levels correlate with low response in patients with taxanes chemotherapy [7]. The gene expression of the group of topoisomerase—topoisomerase 1 (*TOP1*) and 2α (*TOP2α*)—is important for doxorubicin. These enzymes change the topology of DNA and catalyze the unwinding of DNA supercoils and the breaking and stitching of nucleic acid molecules. The expression level of *TOP2α* positively correlates with the efficacy of anthracycline drugs [8]. Several experimental and clinical studies confirm that both the expression of *TOP2α* and the amplification are associated with a worse prognosis. At the same time, such patients are more sensitive to anthracyclines-based therapy, in particular doxorubicin and epirubicin [9].

It is important to note that studies of chromosomal aberrations, in particular, copy number aberrations (CNA) deletions and amplifications, are useful for studying the effect of the presented genes on the neoplasms chemosensitivity. It is well known that allelic deletion of a gene locus can significantly reduce its spontaneous expression and/or its ability to express in response to a stimulus, while amplification is the opposite [10]. It was found that with the deletion of the short arm of chromosome 18 (18p11.32), where the *TYMS* gene is localized, patients are immune to chemotherapy with 5-fluorouracil [6]. Amplification of 16q24.3 (localization of the *TUBB3* gene) is associated with high efficiency of taxanes [11].

Thus, the assessment of the gene expression level before chemotherapy can be useful for choosing the correct and most effective treatment scheme. However, despite a large number of ongoing fundamental and clinical studies, there is no consensus regarding the predictive value of the studied criteria, or the selection of the scheme for breast cancer therapy.

In the present study, we analyzed the association of chemotherapy’s genes expression in breast cancer tissue before and after neoadjuvant chemotherapy with the effect of therapy, as well as indicators of metastatic survival.

## 2. Materials and Methods

### Patients and Treatment

The study involved 97 luminal B breast cancer patients of stages IIA–IIIB (T_1–4_N_0–3_M_0_) with morphologically verified diagnosis, aged 24–68, with the average age being 46.97 ± 1.08 years old (Mean ± SE), who received treatment in the clinics of the Research Institute of Oncology (Tomsk, Russia) in 2006–2020. The research was conducted in accordance with the 1964 Helsinki Declaration (amended in 2013) and the local ethics committee of the institute (protocol 1 dated 14 January 2013), and all patients signed an informed consent for the study. All patients with ‘’Consensus conference on neoadjuvant chemotherapy in carcinoma of the breast, 26–28 April 2003, Philadelphia, Pennsylvania’’ [12] in the neoadjuvant regimen and received 4–8 courses of chemotherapy according to the schemes AC (adriamycin 50 mg/m^2^ and cyclophosphamide 600 mg/m^2^ once every 3 weeks), AT (adriamycin 50 mg/m^2^ and Taxotere 75 mg/m^2^), ACT (adriamycin 50 mg/m^2^, cyclophosphamide 600 mg/m^2^, and Taxotere 75 mg/m^2^), CAX (cyclophosphamide 100 mg/m^2^ intramuscularly, adriamycin 30 mg/m^2^ intravenously, and xeloda 1200 mg/m^2^ orally), or CP (cyclophosphamide 1080 mg/m^2^, cisplatin 135 mg), or monotherapy with Taxotere (100 mg/m^2^ hourly infusion per day). The operation was performed 3–5 weeks after NAC in the amount of radical or subcutaneous mastectomy, radical resection, sectoral resection with axillary lymphadenectomy, or another type of organ-preserving surgery; then, the patients underwent radiation and/or hormonal or targeted therapy (Herceptin in HER2+ status) according to indications. During the entire period, the patients were monitored dynamically. Median follow-up time was 40 months (40.0 ± 2.79). The main clinical and pathological characteristics are presented in Table 1.

We analyzed biopsy tumor samples before treatment (~10 mm^3^ volume), obtained under the control of ultrasound and surgical samples after NAC (~60–70 mm^3^ volume) 3–5 weeks after the last course of neoadjuvant chemotherapy. Tumor samples were placed in an RNAlater solution (Sigma, St. Louis, MO, USA) and stored at –80 °C (after a 24-h incubation at +4 °C) for further DNA isolation.

RNA extraction. Total RNA was isolated from paired samples using the RNeasy Mini kit Plus kit (Qiagen, Germany #51304). The concentration and purity of RNA isolation was evaluated on a NanoDrop-2000 spectrophotometer (Thermo Fisher, Waltham, MA, USA). RNA concentration was 25–100 ng/μL, A_260_/A_280_ = 1.75–1.90, and A_260_/A_230_ = 1.80–2.00. RNA integrity was assessed by capillary electrophoresis on a TapeStation instrument (Agilent Technologies, Santa Clara, CA, USA); DNA fragments had a mass of more than 60 kbp. RIN was 6.6–9.2. To obtain cDNA on an RNA template, a reverse transcription reaction was performed using a RevertAid ™ kit (Thermo Fisher, Waltham, MA, USA) with random hexanucleotides.

Quantitative PCR. The expression level of genes *RRM1*, *ERCC1*, *TOP1*, *TOP2a*, *TUBB3*, *TYMS*, and *GSTP1* was assessed using reverse transcriptase quantitative real-time PCR (RT-qPCR) with original primers and probes using TaqMan technology on a Rotor-Gene-6000 amplifier (Corbett Research, Mortlake, NSW, Australia). PCR was set up in three replicas in a volume of 15 μL containing 250 μM dNTPs (Sibenzyme, Novosibirsk, Russia), 300 nM forward and reverse primers, 200 nM probe, 2.5 mM MgCl2, 19 SE buffer (67 mM Tris—HCl pH 8.8 at 25 °C, 16.6 mM (NH4) 2SO4, and 0.01% Tween-20), 2.5 units of HotStart Taq polymerase (Sibenzyme, Russia), and 50 ng of cDNA. The two-step amplification program included 1 cycle—94 °C, 10 min—pre-denaturation; and 40 cycles—1 step 94 °C, 10 s, and 2 steps 20 s—at a temperature of 60 °C. Two referee genes were used as the referee gene: *GAPDH* (glyceraldehydes-3-phosphatedehydrogenase) and *ACTB* (actin beta), and the level of gene expression was normalized in relation to the expression of the referee genes and measured in arbitrary units. Relative expression was estimated using the Pfaffl method [13]. If the level of gene expression was more than 1 (higher than in normal tissue), then high expression was stated; if the level of gene expression was less than 1 (lower than in normal tissue), then low expression was stated. Primers and probes are presented in Table 2.

DNA extraction. DNA was isolated from 97 samples of tumor tissue using the QIAamp DNA mini Kit (Qiagen, Germany). DNA concentration and purity of isolation were evaluated on a Qubit 4.0 (Thermo Fisher Scientific, USA) from 50 to 250 ng/μL. DNA integrity was assessed by capillary electrophoresis on a TapeStation instrument (Agilent Technologies, USA), and DNA fragments had a mass of more than 60 kbp.

Microarray analysis. Microarray analysis was performed on high-density microarrays (DNA chips) of Affymetrix (USA) CytoScanTM HD Array, which contain 1 million 900 thousand markers non-polymorphic markers for the analysis of copy number aberrations (CNA). Sample preparation, hybridization, and scanning procedures were performed in accordance with the protocol on the Affymetrix GeneChip^®^ Scanner 3000 7G system (Affymetrix, Santa Clara, CA, USA). The Chromosome Analysis Suite 4.3 software (Affymetrix, USA) was used to process the microchipping results, which was specially developed for analyzing the results of microchipping on the CytoScanTM HD Array.

Statistical data processing. Statistical data processing was carried out using the software package Statistica 8.0 (StatSoft Inc., Palo Alto, CA, USA). The Shapiro–Wilk Criterion was used to check the normality of the sample. For each sample, medians and an interquartile range of 25–75% were calculated. To test the hypothesis about the significance of differences between the study groups, the nonparametric Wilcoxon–Mann–Whitney test was used. For the analysis of metastatic-free survival (MFS), the survival curves constructed by the Kaplan–Meier method and the log-rank test were used. The Chi-square test was used to assess differences in frequencies (http://vassarstats.net/index.html, accessed on 2 February 2022). ROC analysis and multivariate Cox analysis were performed using the IBM SPSS Statistics software. As a quantitative interpretation of the ROC analysis, the AUC (Area Under Curve) indicator is given.

## 3. Results

At the first stage of the study, we assessed the relationship between the expression and aberrations of the DNA copy number of the genes of chemosensitivity with the main clinical and pathological parameters (Tables S1 and S2). Significant differences are shown for the *TOP1* gene in the expression level. The postoperative level of this gene is higher in patients with a large primary tumor node (1.34 ± 0.57), compared with patients in the T_1–2_ group (0.85 ± 0.28), with *p =* 0.02. The menstrual status is important for the *TOP2α* gene. In patients with preserved menstrual function, there is a more increased expression of topoisomerase 2α (8.84 ± 2.23) than in postmenopausal patients (4.16 ± 1.44), *p =* 0.05. Only the histological tumor form is associated with the frequency of chromosomal aberrations in genes (Table S2). It was found that the frequency of deletions, in the case of the *ERCC1* gene, is higher in the unicentric form (17.9%, 7/39 cases) than in the multicentric form (3.4%, 2/58 cases), *p =* 0.03. The opposite picture is observed for the *TYMS* gene: deletions were found in 14 out of 58 patients (24.1%) in the multicentric form and in 6 out of 39 patients (15.4%) in the unicentric form. The differences are statistically significant, *p =* 0.03.

**Figure 1:**
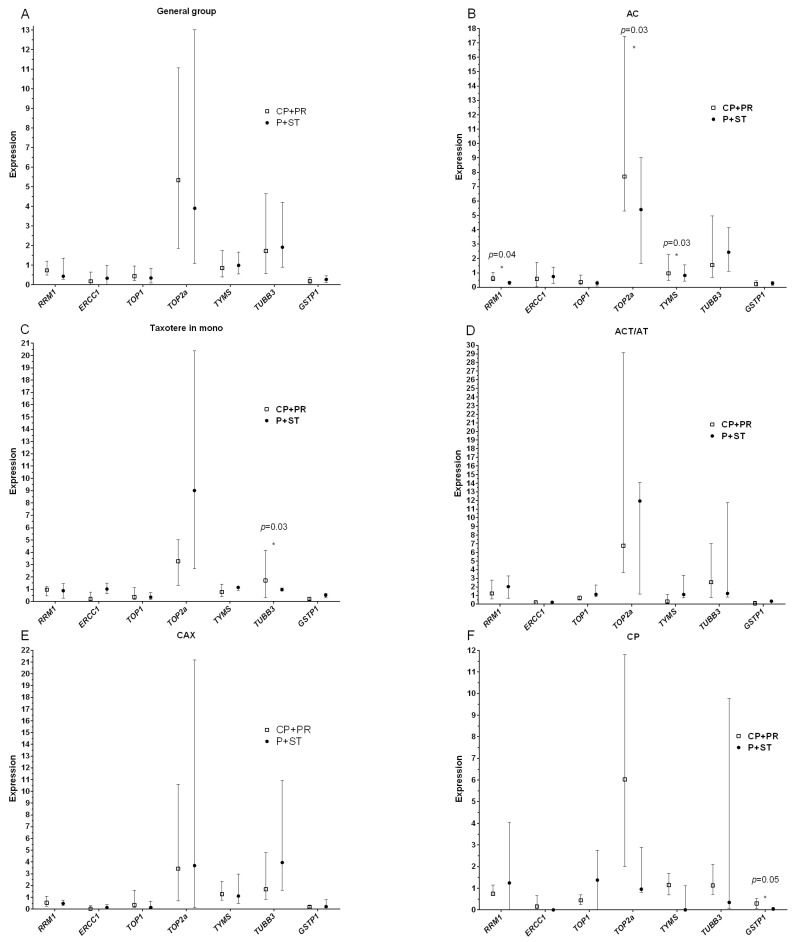
Diagrams of the relationship between the initial level of mRNA expression and the NAC effect in the general group of patients (**A**), depending on the chemotherapy scheme: (**B**)—scheme AC, (**C**)—scheme Taxotere in mono-regimen, (**D**)—scheme AT/ACT, (**E**)—scheme CAX, (**F**)—scheme CP. Note: CR + PR—complete and partial regression; P + ST—progression and stabilization; *—statistically significant result. The figure shows the medians of expression and the interquartile range of 25–75% for each gene in patient groups depending on the effect of NAC.

Then, we analyzed the relationship between the expression of the studied genes and the effect of neoadjuvant chemotherapy (Figure 1).

Statistically significant differences in the level of expression were found for the *RRM1* gene in patients treated with the AC regimen (Figure 1B). The expression of this gene is higher (median: 0.61; percentile 25–75%: 0.44–1.02) in patients with an objective response to treatment (complete and partial regression), compared with patients with stabilization and progression (median: 0.31; percentile 25–75%: 0.16–0.41), with *p =* 0.04. With the same treatment regimen, it was found that high levels of topoisomerase 2α (*TOP2α*) expression, as well as the thymidylate synthase gene (*TYMS*), are associated with an objective response to treatment, *p =* 0.03 for both genes (Figure 1B).

A similar result was shown for the *TUBB3* gene in patients treated with taxotere in mono-regimen (Figure 1B). The expression level was 2.5 times higher in patients with complete and partial regression (median: 1.71; percentile 25–75%: 0.32–4.16 versus median: 0.97; and percentile 25–75%: 0.89–1.11, *p =* 0.03). An interesting result was shown in analyzing the expression of glutathione S-transferase P1, which is involved in the metabolism of platinum drugs, in particular carboplatin and cisplatin. P1 expression is directly related to the clinical response to chemotherapy treatment [14]. According to our results, the high level of *GSTP1* in a tumor biopsy is associated with low efficiency of CP NAC scheme, compared with the group of patients with a low level of expression and objective response to treatment (median: 0.29; percentile 25–75%: 0.07–0.51 versus median: 0.04; percentile 25–75%: 0.00–1.12, *p =* 0.05), (Figure 1F). In other cases, the level of expression of the studied genes was not associated with the effect of neoadjuvant chemotherapy.

Further analysis of the relationship of chromosomal aberrations in the studied genes in patients with breast cancer showed that CNA weakly correlates with the NAC effect (Table 3).

It was found that the presence of *RRM1* deletion in 37.8% of cases determines an objective response to treatment, while in patients with stabilization and progression, the deletion of this gene is observed only in 10.7% of cases (*p =* 0.04). A similar result was established with the CAX chemotherapy. An interesting result was obtained for the *TOP1* gene. The normal copy number of topoisomerase 1 in patients treated with the CAX scheme was associated with a lack of response to treatment in 85.7% of patients (6/7 cases, *p =* 0.03); 50% of patients with a thymidylate synthase deletion responded to the CAX treatment, while in patients with stabilization and progression, no deletions were observed (the relationship was at the level of a pronounced trend, *p =* 0.07) (Table 3).

The presence of *TUBB3* deletion is decisive for the presence of an objective response For the *TUBB3* gene. The frequency of deletions is statistically significantly higher (41/69, 59.4%) in patients with complete and partial regression than in the other group. At the same time, it is important to note that CNA does not affect the effectiveness of treatment in the group of patients treated with taxotere in mono-regimen.

**Figure 2:**
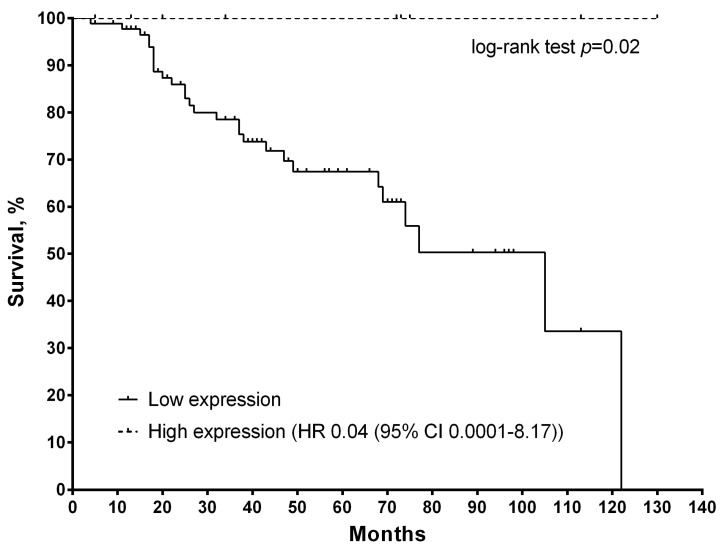
Curves of metastatic survival of breast cancer patients depending on the level of mRNA expression in the surgical material of the *GSTP1* gene (log-rank test *p =* 0.02).

**Figure 3:**
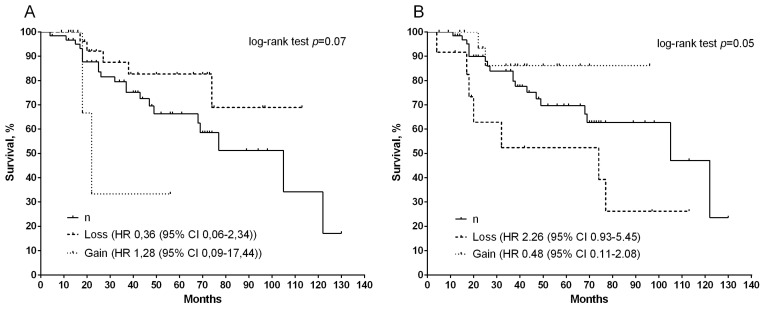
Curves of metastatic survival of patients with breast cancer, depending on CNA gene *RRM1* (**A**) and *GSTP1* (**B**), (log-rank test *p =* 0.07 and *p =* 0.05).

Analysis of metastatic-free survival rates depending on expression, as well as CNA of the studied genes, is presented in Figure 2 and Figure 3. If the level of gene expression was more than 1 (higher than in normal tissue), then high expression was stated; if the level of gene expression was less than 1 (lower than in normal tissue), then low expression was stated. As a result, it was found that statistically significant differences are observed only for the *GSTP1* gene (Figure 2).

In the general group of patients with a *GSTP1* level of more than 1, the 5-year survival rates were 100% versus 68% in the group with low expression (HR 0.04 (95% CI 0.0001–8.17); log-rank test *p =* 0.02).

The study of the expression of other chemosensitivity genes showed an absent relationship, with metastatic survival rates either in the general group of patients or depending on the treatment scheme.

In addition, we also assessed the effect of chromosomal aberrations on metastatic free survival indicators (Figure 3). It was shown that patients with a deletion of the *RRM1* gene have better survival rates than the normal copy number of this gene and amplification at the level of a pronounced trend (Figure 3A), whereas statistically significant differences (log-rank test *p =* 0.05) were shown for *GSTP1*. At the same time, the presence of amplification determines the high survival rate of patients (5-year MFS is 86%), while with a deletion, this indicator slightly exceeds 50% (Figure 3B).

The ROC analysis showed that only the gene *GSTP1* (AUC = 0.677, *p =* 0.01) was significant. None of the remaining genes were significant: *RRM1* (AUC = 0.537, *p =* 0.65); *ERCC1* (AUC = 0.496, *p =* 0.95); *TOP1* (AUC = 0.547, *p =* 0,53); *TOP2a* (AUC = 0.616, *p =* 0,12); *TUBB3* (AUC = 0.604, *p =* 0.16); and *TYMS* (AUC = 0.613, *p =* 0.12).

In addition, a multivariate regression analysis was performed to identify prognostic factors for metastasis-free survival (Table 4).

It was found that the presence of a deletion in the *TYMS* gene and amplification in *GSTP1* are factors that increase the risk of tumor metastasis (HR = 0.17; 95% CI: 0.02–1.03, *p =* 0.05 and HR = 0.48; 95% CI: 0.11–2.08, *p =* 0.04, respectively), whereas *TUBB3* deletion, on the contrary, caused a low risk of metastasis (HR = 5.31; 95% CI: 0.99–28.36, *p =* 0.05), as well as a high level of *TOP2α* gene expression (HR = 3.29; 95% CI: 1.15–9.41, *p =* 0.02), (Table 4). Clinical and pathological parameters do not affect the risk of metastases.

## 4. Discussion

To date, it has been established that the expression and/or co-expression of genes for chemosensitivity in tumor tissues is closely related to chemoresistance and prognosis in patients with breast cancer [2]. According to these works, gene expression, although it showed a high relationship with the effectiveness of treatment, is a variable value. Therefore, it is necessary to assess additional parameters of the studied genes of chemosensitivity. In our study, in addition to assessing the expression of genes for chemosensitivity, we analyzed the aberrations of the DNA copy number. It was found that the presence of *TUBB3* and *RRM1* deletion in tumor biopsy material is associated with more effective treatment. Besides this, the presence of a deletion of *GSTP1* and *RRM1* determines higher MFS values. Our data are consistent with the literature data.

Ribonucleotide reductase consists of two subunits, *RRM1* and *RRM2*, and is an enzyme that limits the rate of DNA synthesis [15]. The *RRM1* gene is the main target for gemcitabine. It has been shown that high expression of *RRM1* is associated with resistance to this chemotherapy drug in a lung tumor [16]. At the same time, we showed in our study that increased *RRM1* expression in patients treated with the AC scheme and deletion in patients treated with the CAX scheme determines the presence of objective response to treatment. In another study, the authors showed that *RRM1* copy number aberrations (deletions and amplifications) were observed in 15.9% and 13.6% of patients, respectively. Their presence was associated with a decrease in survival rates (HR = 1.72, 95% CI = 1.05–2.79, *p* = 0.03) [17]. The high *TYMS* expression and low *RRM1* significantly correlate with sensitivity to gemcitabine [6]. However, in other clinical studies of breast cancer [18], lung cancer [19], and colorectal cancer [20], patients with low *TYMS* expression showed better chemotherapy response and higher survival rates.

*TUBB3* is the main component of microtubules, which is a structural component of the division spindle and cytoskeleton [21]. Upregulation of *TUBB3* expression can destabilize microtubules and inhibit taxanes [7], which has been confirmed in various types of cancer, including breast cancer [22,23], lung cancer, ovarian cancer, prostate cancer, stomach cancer, and pancreatic cancer [24]. We have shown that a high level of *TUBB3* expression is a favorable predictive marker in patients treated with taxotere in mono-regimen (*p =* 0.01).

Patients with low *TOP2α* expression treated with anthracycline-containing regimens showed no response to treatment, and low survival rates [8,25]. This is consistent with our results. Positive expression of *TOP2α* is associated with low rates of overall and disease-free survival (*p =* 0.024 and *p =* 0.039, respectively) [26]. It is important to note that the predictive and prognostic significance of changes in the *TOP2α* copy number remains unclear. It has been shown that the change in the number of *TOP2α* copies is a rare genetic event (the frequency of amplifications and deletions is 9.8% and 2.7%, respectively) and does not have prognostic value [27].

The expression of *GSTP1* is higher in the group of chemoresistant breast tumor cells, which may be reflected in the therapeutic response of patients to treatment [4]. Thus, it was found that patients with low or absent *GSTP1* expression more often had an objective response to NAC with docetaxel (*p =* 0.005) and paclitaxel (*p =* 0.006) [28]. In addition, various genetic variants of *GSTP1* may play an important role in the effectiveness of platinum-based chemotherapy [5,29], as shown in our work: an initially high level of expression of this gene is associated with a low efficacy of chemotherapy according to the CP scheme (*p =* 0.05). However, interestingly, *GSTP1* overexpression after NAC is associated with 100% MFS (log-rank test *p =* 0.02). Other authors found that the presence of another disorder in the *GSTP1* gene (in particular, methylation) in tumor tissue closely correlates with the clinical and pathological features of breast cancer, which indicates the possibility of using this gene for tumor diagnosis and prognosis [30].

In a recent work, it was shown that the expression levels of *ERCC1*, *TYMS,* and *TOP2α* were significantly associated with clinical and pathological parameters: menopausal status, tumor size, lymph node metastasis, hormone receptor status, triple-negative status, Ki-67 index, and epidermal growth factor receptor [31]. With respect to *ERCC1* gene, the higher intensity was significantly related to T_1_ tumor (mean rank: 64.79 > 42.26, *p* < 0.001), ER-positive (mean rank: 54.98 > 37.41, *p* = 0.002), PR-positive (mean rank: 58.35 > 39.05, *p* < 0.001) and Ki-67 < 20% (mean rank: 66.00 > 44.30, *p* = 0.001). In terms of *TYMS* gene, patients with Ki-67 ≥ 20% exhibited higher expression level (mean rank: 52.76 > 35.40, *p* = 0.011). The expression *TOP2α* intensity was higher in the premenopausal group (mean rank: 54.28 > 42.90, *p* = 0.040) and lymph node metastasis group (mean rank: 55.19 > 43.64, *p* = 0.037). Similar results were observed in Ki-67 ≥ 20% group (mean rank: 53.63 > 32.26, *p* = 0.001). Our analysis of the relationship of expression showed that the postoperative level of *TOP1* gene is higher in patients with a large primary tumor node (1.34 ± 0.57) than in patients in the T_1–2_ group (0.85 ± 0.28), with *p =* 0.02. The result of the analysis of the expression of *TOP2α* is consistent with the results of this study: in patients with preserved menstrual function, there is greater expression of topoisomerase 2α (8.84 ± 2.23) than in postmenopausal patients (4.16 ± 1.44), *p =* 0.05. For other genes, we did not establish a statistically significant relationship between expression and clinical and pathological parameters of patients with breast cancer.

As a result of the ROC-analysis, it was shown that the genetic results of expression showed no predictive power, except for the expression of the *GSTP1* gene (AUC = 0.677, *p =* 0.01), which is consistent with the results of the analysis by the Kaplan–Meier method and the log-rank test. In summary, the results of the analysis in the presented study indicate that the expression of the studied genes has controversial predictive potential. However, further large-scale prospective studies with multivariate predictive analysis, in addition to control samples and the validation of a standardized method, are needed to elucidate the usefulness of these biomarkers in breast cancer.

## Figures and Tables

**Table 1:** The main clinical and pathological parameters of patients.

Clinical and Pathological Parameter		The Number of Patients, abs.n. (%)
Age	≤45	44 (45.4)
	>45	53 (54.6)
Menstrual status	Premenopause	51 (52.6)
	Postmenopause	46 (47.4)
Tumor size	T_1_	15 (15.5)
	T_2_	71 (73.2)
	T_3_	5 (5.2)
	T_4_	6 (6.2)
Lymphogenous metastasis	N_0_	40 (41.2)
	N_1_	44 (45.4)
	N_2_	6 (6.2)
	N_3_	7 (7.2)
Histological form	Unicentric	39 (40.2)
	Multicentric	58 (59.8)
Histological type	Invasive ductal carcinoma	54 (55.7)
	Invasive lobular carcinoma	43 (44.3)
NAC regimen	CAX	19 (19.6)
	AC	30 (30.9)
	Taxotere in mono	21 (21.6)
	AT/ACT	16 (16.5)
	CP	11 (11.3)
NAC effect	Complete regression	11 (11.3)
	Partial regression	58 (59.8)
	Stabilization	25 (25.8)
	Progression	3 (3.1)

**Table 2:** Sequence of primers and probes.

Gene	Amplicon (bp)	Sequence
*GAPDH*	124 bp	F 5′-gccagccgagccacatc-3′
		R 5′-ggcaacaatatccactttaccaga-3′
		Probe 5′-cgcccaatacgaccaaatccg-3′
*RRM1*	94 bp	F 5′-actaagcaccctgactatgctatcc-3′
		R 5′-cttccatcacatcactgaacacttt-3′
		Probe 5′-cagccaggatcgctgtctctaacttgca-3′
*ERCC1*	121 bp	F 5′-ggcgacgtaattcccgacta-3′
		R 5′-agttcttccccaggctctgc-3′
		Probe 5′-accacaacctgcacccagactacatcca-3′
*TOP1*	97 bp	F 5′-ggcgagtgaatctaaggataatgaa -3′
		R 5′- tggatatcttaaagggtacagcgaa -3′
		Probe 5′-accattttcccatcatcctttgttctgagc -3′
*TOP2α*	75 bp	F 5′-agtcgctttcagggttcttgag-3′
		R 5′-tttcatttacaggctgcaatgg-3′
		Probe 5′-cccttcacgaccgtcaccatgga-3′
*TUBB3*	71 bp	F 5′-gggccaagttctgggaagtc-3′
		R 5′-cgagtcgcccacgtagttg-3′
		Probe 5′-atgagcatggcatcgaccccagc-3′
*TYMS*	91 bp	F 5′-tctggaagggtgttttgga-3′
		R 5′-tcccagattttcactccctt-3′
		Probe 5′-tctttagcatttgtggatcccttga-3′
*GSTP1*	84 bp	F 5′-ctggtggacatggtgaatgac-3′
		R 5′-cttgcccgcctcatagttg-3′
		Probe 5′-aggacctccgctgcaaatacatctc-3′

Note: all probes—FAM→BHQ1; NM—RNA sequence number in NCBI nucleotide database (http://www.ncbi.nlm.nih.gov/nuccore, accessed on 2 February 2022); bp—base pair; F—forward primer; R—reversed praimer; Probe—probe.

**Table 3:** Frequency of chromosomal aberrations in genes chemosensitivity depending on the effect and NAC.

Genes	CNA	General Group		CAX		AC		Taxotere in Mono		ACT/AT		CP	
		CR + PR	P + ST	CR + PR	P + ST	CR + PR	P + ST	CR + PR	P + ST	CR + PR	P + ST	CR + PR	P + ST
*RRM1*	Loss	24 (34.8)	3 (10.7)	6 (50.0)	0 (0.0)	9 (45.0)	1 (10.0)	3 (18.8)	1 (20.0)	4 (30.8)	0 (0.0)	2 (25.0)	1 (33.3)
	n	42 (60.9)	22 (78.6)	6 (50.0)	6 (85.7)	10 (50.0)	8 (80.0)	13 (81.3)	4 (80.0)	8 (61.5)	2 (66.7)	5 (62.5)	2 (66.7)
	Gain	3 (4.3)	3 (10.7)	0 (0.0)	1 (14.3)	1 (5.0)	1 (10.0)	0 (0.0)	0 (0.0)	1 (7.7)	1 (33.3)	1 (12.5)	0 (0.0)
	*p*-level	**0.04**		**0.04**		0.15		1		0.32		0.80	
*ERCC1*	Loss	6 (8.7)	4 (14.3)	3 (25.0)	1 (14.3)	1 (5.0)	1 (10.0)	1 (6.3)	0 (0.0)	0 (0.0)	0 (0.0	1 (12.5)	2 (66.7)
	n	60 (87.0)	23 (82.1)	9 (75.0)	6 (85.7)	18 (90.0)	9 (90.0)	15 (93.8)	4 (80.0)	11 (84.6)	3 (100.0)	7 (87.5)	1 (33.3)
	Gain	3 (4.3)	1 (3.6)	0 (0.0)	0 (0.0)	1 (5.0)	0 (0.0)	0 (0.0)	1 (20.0)	2 (15.4)	0 (0.0)	0 (0.0)	0 (0.0)
	*p*-level	0.70		0.85		0.68		0.16		1		0.15	
*TOP1*	Loss	3 (4.3)	0 (0.0)	3 (25.0)	0 (0.0)	0 (0.0)	0 (0.0)	0 (0.0)	0 (0.0)	0 (0.0)	0 (0.0)	0 (0.0)	0 (0.0)
	n	42 (60.9)	23 (82.1)	3 (25.0)	6 (85.7)	10 (50.0)	8 (80.0)	14 (87.5)	5 (100.0)	8 (61.5)	2 (66.7)	7 (87.5)	2 (66.7)
	Gain	24 (34.8)	5 (17.9)	6 (50.0)	1 (14.3)	10 (50.0)	2 (20.0)	2 (12.5)	0 (0.0)	5 (38.5)	1 (33.3)	1 (12.5)	1 (33.3)
	*p*-level	0.10		**0.03**		0.28		1		1		0.99	
*TOP2a*	Loss	14 (20.3)	8 (28.6)	3 (25.0)	1 (14.3)	3 (15.0)	3 (30.0)	3 (18.8)	2 (40.0)	0 (0.0)	0 (0.0)	5 (62.5)	2 (66.7)
	n	38 (55.1)	15 (53.6)	4 (33.3)	5 (71.4)	11 (55.0)	4 (40.0)	11 (68.8)	3 (60.0)	9 (69.2)	2 (66.7)	3 (37.5)	1 (33.3)
	Gain	17 (24.6)	5 (17.9)	5 (41.7)	1 (14.3)	6 (30.0)	3 (30.0)	2 (12.5)	0 (0.0)	4 (30.8)	1 (33.3)	0 (0.0)	0 (0.0)
	*p*-level	0.60		0.26		0.59		0.49		1		1	
*TYMS*	Loss	21 (30.4)	4 (14.3)	6 (50.0)	0 (0.0)	7 (35.0)	2 (20.0)	4 (25.0)	0 (0.0)	3 (23.1)	0 (0.0)	1 (12.5)	2 (66.7)
	n	45 (65.2)	21 (75.0)	5 (41.7)	6 (85.7)	13 (65.0)	7 (70.0)	12 (75.0)	4 (80.0)	8 (61.5)	3 (100.0)	7 (87.5)	1 (33.3)
	Gain	3 (4.3)	3 (10.7)	1 (8.3)	1 (14.3)	0 (0.0)	1 (10.0)	0 (0.0)	1 (20.0)	2 (15.4)	0 (0.0)	0 (0.0)	0 (0.0)
	*p*-level	0.16		0.07		0.28		0.10		0.43		0.15	
*TUBB3*	Loss	41 (59.4)	4 (22.2)	5 (41.7)	3 (42.9)	10 (50.0)	6 (60.0)	13 (81.3)	4 (80.0)	10 (76.9)	1 (33.3)	3 (37.5)	0 (0.0)
	n	25 (36.2)	13 (72.2)	5 (41.7)	4 (57.1)	10 (50.0)	4 (40.0)	3 (18.8)	1 (20.0)	1 (7.7)	1 (33.3)	4 (50.0)	3 (100.0)
	Gain	3 (4.3)	1 (5.6)	2 (16.7)	0 (0.0)	0 (0.0)	0 (0.0)	0 (0.0)	0 (0.0)	2 (15.4)	1 (33.4)	1 (12.5)	0 (0.0)
	*p*-level	**0.01**		0.49		0.87		1		0.30		0.30	
*GSTP1*	Loss	7 (10.1)	4 (14.3)	3 (25.0)	2 (28.6)	2 (10.0)	1 (10.0)	0 (0.0)	1 (20.0)	2 (15.4)	0 (0.0)	0 (0.0)	0 (0.0)
	n	46 (66.7)	20 (71.4)	5 (41.7)	5 (71.4)	15 (75.0)	7 (70.0)	12 (75.0)	4 (80.0)	7 (53.8)	2 (66.7)	7 (87.5)	2 (66.7)
	Gain	16 (23.2)	4 (14.3)	4 (33.3)	0 (0.0)	3 (15.0)	2 (20.0)	4 (25.0)	0 (0.0)	4 (30.8)	1 (33.3)	1 (12.5)	1 (33.3)
	*p*-level	0.56		0.21		0.94		0.10		0.76		0.99	

Note: CR+PR—complete and partial regression; P+ST—progression and stabilization. Statistically significant differences are in bold.

**Table 4:** Multivariate Cox regression analysis for metastasis-free survival of patients with breast cancer.

Factor	MFS	
	HR (95% CI)	*p*-Value
Clinical and pathological parameter		
Age		
≤45	1.00	
>45	2.23 (0.46–10.84)	0.32
Tumor size		
T_1–2_	1.00	
T_3–4_	4.45 (1.91–10.34)	0.24
Lymphogenous metastasis		
N_0_	1.00	
N_1_	0.93 (0.22–3.95)	0.92
N_2_	7.20 (0.91–56.74)	0.06
N_3_	6.57 (0.90–48.16)	0.06
Menstrual status		
Premenopause	1.00	
Postmenopause	0.61 (0.13–2.78)	0.52
Histological type		
Invasive ductal carcinoma	1.00	
Invasive lobular carcinoma	0.83 (0.20–3.41)	0.79
Histological form		
**Unicentric**	1.00	
**Multicentric**	3.07 (0.62–15.15)	0.17
NAC effect		
Complete/Partial regression	1.00	
Stabilization/Progression	2.16 (0.61–7.69)	0.23
Copy number aberrations		
*RRM1*		
n	1.00	
Loss	0.36 (0.06–2.34)	0.29
Gain	1.28 (0.09–17.44)	0.85
*ERCC1*		
n	1.00	
Loss	2.23 (0.26–18.96)	0.46
Gain	0.98 (0.06–16.34)	0.99
*TOP1*		
n	1.00	
Loss	0.40 (0.004–40.54)	0.77
Gain	1.46 (0.10–20.69)	0.38
*TOP2α*		
n	1.00	
Loss	3.29 (0.59–18.52)	0.18
Gain	0.39 (0.05–2.81)	0.35
*TYMS*		
n	1.00	
Loss	0.17 (0.02–1.03)	**0.05**
Gain	1.36 (0.09–18.92)	0.82
*TUBB3*		
n	1.00	
Loss	5.31 (0.99–28.36)	**0.05**
Gain	0.73 (0.03–17.72)	0.84
*GSTP1*		
n	1.00	
Loss	2.26 (0.93–5.45)	0.69
Gain	0.48 (0.11–2.08)	**0.04**
Expression		
*RRM1*		
Low expression	1.00	
High expression	1.18 (0.15–9.44)	0.88
*ERCC1*		
Low expression	1.00	
High expression	0.76 (0.17–3.44)	0.72
*TOP1*		
Low expression	1.00	
High expression	5.09 (0.46–55.83)	0.18
*TOP2α*		
Low expression	1.00	
High expression	3.29 (1.15–9.41)	**0.02**
*TYMS*		
Low expression	1.00	
High expression	1.21 (0.21–6.89)	0.83
*TUBB3*		
Low expression	1.00	
High expression	0.37 (0.08–1.76)	0.21
*GSTP1*		
Low expression	1.00	
High expression	0.09 (0.003–2.86)	0.17

Note: Statistically significant differences are in bold.

## Data Availability

Database registration certificate RU 2015621620 30/10/2015 Tsyganov, M.M., Ibragimova, M.K., Deryusheva, I.V., Kazantseva, P.V., Slonimskaya, E.M., Litvyakov, N.V. Database of normal genetic variability of breast tumors and the level of expression of chemoresistance genes.

## Supplementary Materials

The following supporting information can be downloaded at: https://www.mdpi.com/article/10.3390/diagnostics12020405/s1, Table S1: Relationship between the expression of genes of chemosensitivity with the main clinical and pathological parameters (median/percentile 25–75%); Table S2: The frequency of chromosomal aberrations in the genes of chemosensitivity, depending on the effect and scheme of NAC.
