# Supplementary material for: Predictive and Prognostic Significance of mRNA Expression and DNA Copies Aberrations of ERCC1, RRM1, TOP1, TOP2A, TUBB3, TYMS, and GSTP1 Genes in Patients with Breast Cancer
Source: Diagnostics (Basel). 2022 Feb 4;12(2):405. doi: 10.3390/diagnostics12020405 (PMC8871321; doi:10.3390/diagnostics12020405)
Supplement: Supplementary file 1 [file diagnostics-12-00405-s001.zip › diagnostics-1552098-SI.pdf]

Supplement 1

Table S1 - Relationship between the expression of genes of chemosensitivity with the main clinical and pathological parameters (median/percentile 25-75%).

Clinical and pathological parameter		Age			Tumor size			Lymphogenous metastasis			Histological form			Menstrual status		
		<45	>45	*p*-level	T_{1-2}	T_{3-4}	*p*-level	N_0	N_{1-3}	*p*-level	Uni	Multi	*p*-level	Pre-	Post-	*p*-level
Expression before NAC	*RRM1*	1.01±0.14	1.16±0.24	0.46	1.08±0.16	1.23±0.40	0.44	1.23±0.28	0.99±0.14	0.91	0.87±0.14	1.25±0.22	0.56	0.97±0.13	1.20±0.25	0.56
	ERCC1	0.56±0.14	0.58±0.12	0.67	0.57±0.10	0.55±0.20	0.68	0.57±0.18	0.57±0.10	0.77	0.58±0.15	0.56±0.12	0.98	0.66±0.13	0.47±0.13	0.41
	TOP1	0.82±0.17	6.47±5.71	0.06	4.36±3.56	0.70±0.18	0.65	0.80±0.19	6.09±5.31	0.74	8.58±7.76	0.77±0.14	0.38	6.74±5.94	0.77±0.17	0.69
	TOP2a	15.54±4.20	12.33±3.75	0.38	13.80±3.07	13.46±5.71	0.83	13.81±4.93	13.74±3.30	0.87	9.13±1.58	16.94±4.54	0.87	14.50±3.69	12.94±4.27	0.91
	TYMS	1.29±0.22	1.39±0.20	0.61	1.32±0.15	1.56±0.54	0.97	1.54±0.24	1.22±0.18	0.18	1.62±0.25	1.16±0.17	0.14	1.43±0.22	1.25±0.19	0.86
	TUBB3	3.72±0.64	3.01±0.47	0.59	3.20±0.39	4.32±1.47	0.68	3.61±0.66	3.13±0.47	0.83	2.68±0.41	3.77±0.58	0.83	3.56±0.53	3.07±0.56	0.38
	GSTP1	0.44±0.13	0.77±0.32	0.12	0.61±0.21	0.70±0.46	0.39	0.55±0.22	0.67±0.28	0.39	0.99±0.44	0.37±0.10	0.06	0.75±0.32	0.47±0.18	0.81
Expression after NAC	*RRM1*	0.97±0.28	0.85±0.17	0.88	0.84±0.16	1.26±0.42	0.11	0.82±0.26	0.94±0.17	0.2	0.69±0.09	1.08±0.27	0.76	0.90±0.20	0.88±0.22	0.51
	ERCC1	0.76±0.31	1.44±0.84	0.69	1.31±0.62	0.27±0.15	0.25	0.48±0.14	1.59±0.85	0.13	1.78±1.10	0.63±0.23	0.14	1.80±0.99	0.46±0.13	0.07
	TOP1	0.80±0.24	0.96±0.38	0.84	0.85±0.28	1.34±0.57	**0.02**	0.51±0.11	1.13±0.40	0.94	1.01±0.50	0.81±0.17	0.12	1.17±0.46	0.58±0.11	0.49
	TOP2a	10.73±3.33	4.57±1.12	0.17	6.29±1.42	10.29±5.80	0.63	7.77±3.06	6.11±1.37	0.42	6.90±2.07	6.54±1.93	0.71	8.84±2.23	4.16±1.44	**0.05**
	TYMS	1.76±0.70	2.13±0.80	0.9	1.76±0.59	3.99±2.02	0.08	2.72±1.39	1.59±0.43	0.66	1.27±0.29	2.68±1.07	0.45	1.830.50±	2.20±1.12	0.41
	TUBB3	3.87±1.29	3.32±0.62	0.85	3.47±0.65	3.92±1.46	0.44	3.93±0.98	3.28±0.77	0.29	3.79±0.80	3.25±0.90	0.39	4.27±0.90	2.61±0.73	0.06
	GSTP1	0.49±0.10	0.53±0.07	0.41	0.54±0.06	0.38±0.15	0.32	0.52±0.09	0.52±0.08	0.78	0.54±0.09	0.50±0.07	0.83	0.56±0.09	0.47±0.07	0.87

Note: Histological form: Uni - unicentric. Multi - multicentric; Menstrual status: Pre-premenopause. Post-postmenopause. Statistically significant differences are in bold.

Table S2 - The frequency of chromosomal aberrations in the genes of chemosensitivity, depending on the effect and scheme of NAC.

Genes	CNA	Age		Tumor size		Lymphogenous metastasis		Histological form		Menstrual status	
		≤45	>45	T_{1-2}	T_{3-4}	N_0	N_{1-3}	Uni	Multi	Pre-	Post-
RRM1	Loss	15 (34.1)	12 (22.6)	23 (26.7)	4 (36.4)	9 (22.5)	18 (31.6)	13 (33.3)	14 (24.1)	15 (29.4)	12 (26.1)
	n	27 (61.4)	37 (69.8)	58 (67.4)	6 (54.5)	27 (67.5)	37 (64.9)	25 (64.1)	39 (67.2)	34 (66.7)	30 (65.2)
	Gain	2 (4.5)	4 (7.5)	5 (5.8)	1 (9.1)	4 (10.0)	2 (3.5)	1 (2.6)	5 (8.6)	2 (3.9)	4 (8.7)
	p-level	0.4		0.69		0.31		0.34		0.60	
ERCC1	Loss	3 (6.8)	7 (13.2)	10 (11.6)	0 (0.0)	5 (12.5)	5 (8.8)	5 (12.8)	5 (8.6)	3 (5.9)	7 (15.2)
	n	38 (86.4)	45 (84.9)	72 (83.7)	11 (100.0)	35 (87.5)	48 (84.2)	33 (84.6)	50 (86.2)	45 (88.2)	38 (82.6)
	Gain	3 (6.8)	1 (1.9)	4 (4.7)	0 (0.0)	0 (0.0)	4 (7.0)	1 (2.6)	3 (5.2)	3 (5.9)	1 (2.2)
	p-level	0.30		0.35		0.20		0.67		0.22	
TOP1	Loss	1 (2.3)	2 (3.8)	3 (3.5)	0 (0.0)	2 (5.0)	1 (1.8)	1 (2.6)	2 (3.4)	1 (2.0)	2 (4.3)
	n	29 (65.9)	36 (67.9)	57 (66.3)	8 (72.7)	23 (57.5)	42 (73.7)	27 (69.2)	38 (65.5)	36 (70.6)	29 (63.0)
	Gain	14 (31.8)	15 (28.3)	26 (30.2)	3 (27.3)	15 (37.5)	14 (24.6)	11 (28.2)	18 (31.1)	14 (27.5)	15 (32.6)
	p-level	0.86		0.79		0.21		0.91		0.64	
TOP2a	Loss	13 (29.5)	9 (17.0)	19 (22.1)	3 (27.3)	7 (17.5)	15 (26.3)	12 (30.8)	10 (17.2)	**15 (29.4)**	**7 (15.2)**
	n	23 (52.3)	30 (56.6)	47 (54.7)	6 (54.5)	20 (50.0)	33 (57.9)	20 (51.3)	33 (56.9)	**30 (58.8)**	**23 (50.0)**
	Gain	8 (18.2)	14 (26.4)	20 (23.3)	2 (18.2)	13 (32.5)	9 (15.8)	7 (17.9)	15 (25.9)	**6 (11.8)**	**16 (34.8)**
	p-level	0.29		0.89		0.13		0.26		**0.01**	
TYMS	Loss	13 (29.5)	12 (22.6)	22 (25.6)	3 (27.3)	7 (17.5)	18 (31.6)	8 (20.5)	17 (29.3)	15 (29.4)	10 (21.7)
	n	29 (65.9)	37 (69.8)	58 (67.4)	8 (72.7)	29 (72.5)	37 (64.9)	27 (69.2)	39 (67.2)	34 (66.7)	32 (69.6)
	Gain	2 (4.5)	4 (7.5)	6 (7.0)	0 (0.0)	4 (10.0)	2 (3.5)	4 (10.3)	2 (3.4)	2 (3.9)	4 (8.7)
	p-level	0.65		0.66		0.16		0.29		0.47	
TUBB3	Loss	26 (59.1)	29 (54.7)	49 (57.0)	6 (54.5)	23 (57.5)	32 (56.1)	23 (59.0)	32 (55.2)	29 (56.9)	26 (56.5)
	n	15 (34.1)	21 (39.6)	32 (37.2)	4 (36.4)	14 (35.0)	22 (38.6)	15 (38.5)	21 (36.2)	20 (39.2)	16 (34.8)
	Gain	3 (6.8)	3 (5.7)	5 (5.8)	1 (9.1)	3 (7.5)	3 (5.3)	1 (2.6)	5 (8.6)	2 (3.9)	4 (8.7)
	p-level	0.84		0.91		0.86		0.47		0.60	
GSTP1	Loss	5 (11.4)	6 (11.3)	9 (10.5)	2 (18.2)	4 (10.0)	7 (12.3)	4 (10.3)	7 (12.1)	6 (11.8)	5 (10.9)
	n	30 (68.2)	36 (67.9)	59 (68.6)	7 (63.6)	29 (72.5)	37 (64.9)	25 (64.1)	41 (70.7)	36 (70.6)	30 (65.2)
	Gain	9 (20.5)	11 (20.8)	18 (20.9)	2 (18.2)	7 (17.5)	13 (22.8)	10 (25.6)	10 (17.2)	9 (17.6)	11 (23.9)
	p-level	1		0.74		0.72		0.60		0.74	

Note: Histological form: Uni - unicentric. Multi - multicentric; Menstrual status: Pre-premenopause. Post-postmenopause. Statistically significant differences are in bold.
